# Microsite conditions in retrogressive thaw slumps may facilitate increased seedling recruitment in the Alaskan Low Arctic

**DOI:** 10.1002/ece3.4882

**Published:** 2019-01-28

**Authors:** Diane Christine Huebner, Marion Syndonia Bret‐Harte

**Affiliations:** ^1^ Department of Biology & Wildlife University of Alaska Fairbanks Fairbanks Alaska; ^2^ Institute of Arctic Biology University of Alaska Fairbanks Fairbanks Alaska

**Keywords:** arctic, chronosequence, recruitment, retrogressive thaw slumps, seedbank, thermokarst

## Abstract

In Low Arctic tundra, thermal erosion of ice‐rich permafrost soils (thermokarst) has increased in frequency since the 1980s. Retrogressive thaw slumps (RTS) are thermokarst disturbances forming large open depressions on hillslopes through soil wasting and vegetation displacement. Tall (>0.5 m) deciduous shrubs have been observed in RTS a decade after disturbance. RTS may provide conditions suitable for seedling recruitment, which may contribute to Arctic shrub expansion. We quantified in situ seedling abundance, and size and viability of soil seedbanks in greenhouse trials for two RTS chronosequences near lakes on Alaska's North Slope. We hypothesized recent RTS provide microsites for greater recruitment than mature RTS or undisturbed tundra. We also hypothesized soil seedbanks demonstrate quantity–quality trade‐offs; younger seedbanks contain smaller numbers of mostly viable seed that decrease in viability as seed accumulates over time. We found five times as many seedlings in younger RTS as in older RTS, including birch and willow, and no seedlings in undisturbed tundra. Higher seedling counts were associated with bare soil, warmer soils, higher soil available nitrogen, and less plant cover. Seedbank viability was unrelated to size. Older seedbanks were larger at one chronosequence, with no difference in percent germination. At the other chronosequence, germination was lower from older seedbanks but seedbank size was not different. Seedbank germination was positively associated with in situ seedling abundance at one RTS chronosequence, suggesting postdisturbance revegetation from seedbanks. Thermal erosion may be important for recruitment in tundra by providing bare microsites that are warmer, more nutrient‐rich, and less vegetated than in undisturbed ground. Differences between two chronosequences in seedbank size, viability, and species composition suggest disturbance interacts with local conditions to form seedbanks. RTS may act as seedling nurseries to benefit many Arctic species as climate changes, particularly those that do not produce persistent seed.

## 1 INTRODUCTION

Climate warming in the Arctic is likely to increase the frequency of landscape disturbance (IPCC, 2014), resulting in increased opportunities for seedling recruitment. Seedling recruitment in tundra communities is considered infrequent compared to clonal growth of established individuals (Eriksson, 1989; Rowe, 1983), due to short growing seasons and lack of suitable microsites. Disturbances such as tundra fires (Bret‐Harte et al., 2013), frost boils (Sutton, Hermanutz, & Jacobs, 2018), and rodent activity (Nystuen, Evju, Rusch, Graae, & Eide, 2014) can stimulate seedling recruitment by reducing competition and altering microsites and may ultimately influence the structure of vegetation communities (Chambers, 1995).

In high‐stress environments, germination and establishment are likely the most limiting phases of a plant's ability to colonize an area (Alsos et al., 2007; Grime, 1977). Seeds in northern environments typically break dormancy after a cold period, and seedlings must rapidly establish following snowmelt (Billings & Mooney, 1968). Contact with bare soil may be more optimal for root penetration than dense litter layers (Chapin et al., 2006; Douglas, 1995). Recruitment thus depends on seasonally short windows of suitable microsites and viable seed (Eriksson & Fröborg, 1996). The relationship of increased seedling success in Arctic tundra to disturbance and high‐quality microsites has been supported in other research (Gough, 2006; Graae et al., 2011; Milbau, Shevtsova, Olser, Mooshammer, & Graae, 2013; Munier, Hermanutz, Jacobs, & Lewis, 2010; Nystuen et al., 2014; Sutton et al., 2018). Increased recruitment can lead to populations with novel combinations of genes that could help plant species adapt to rapid change (Petit, 2004).

**Figure 1:**
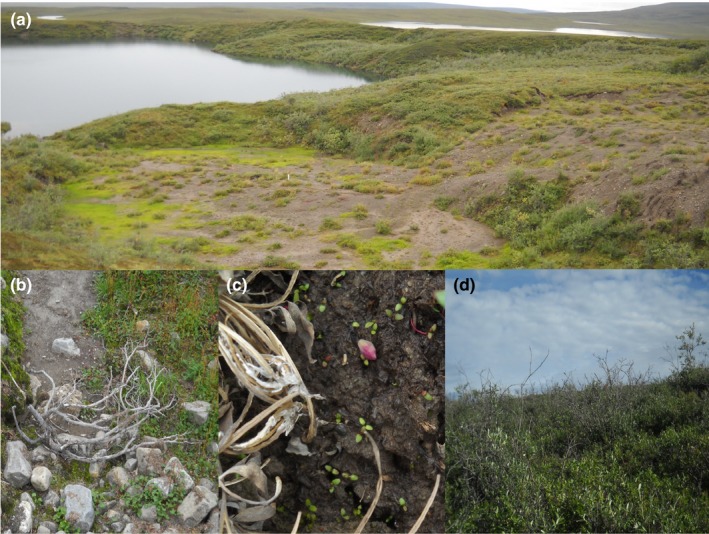
(a) Overview of a young (1–10 years old) retrogressive thaw slump (RTS) on the south shore of lake NE‐14 in the Alaskan Low Arctic, (b) dead shrub in RTS chute at lake I‐minus 1 caused by mass soil wasting, (c) seedlings on bare soil in young RTS at lake I‐minus 1, and (d) tall shrubs in old (≥30 years old) RTS at NE‐14

Thermal erosion is one disturbance likely to influence tundra plant communities. Since the 1980s, thermal erosion of ice‐rich permafrost soils has been observed with increasing frequency (Belshe, Schuur, & Grosse, 2013; Bowden et al., 2008). Retrogressive thaw slumps (RTS) are areas of progressive ground collapse due to the melting of subsurface ice; on Alaska's North Slope, they have been observed on hillslopes and lake shores in the northern foothills of the Brooks Range (Bowden et al., 2008; Gooseff, Balser, Bowden, & Jones, 2009), forming depressions many square meters in area due to mass soil wasting (Figure 1a,b). Time‐lapse photography of two recent North Slope RTS, the 2010 Horn Lake thermokarst (Godsey, Gooseff, & Lewcowicz, 2010), and the 2014 Wolverine Lake thermokarst (Dobkowski, 2014) documented the displacement of entire vegetation communities through mass soil wasting within a single summer.

Concurrent with increasing thermal erosion, aerial surveys of Alaska's North Slope over 50 years have photographically documented the expansion of deciduous woody shrubs in Arctic tundra (IPCC, 2014; Sturm, Racine, & Tape, 2001). Potential feedbacks of a shrubbier Arctic to ecosystem processes include reduced albedo and increased evapotranspiration by shrubs compared to tussock tundra, which can result in greater heat retention (Chapin, 2005; Euskirchen, McGuire, Chapin, Yi, & Thompson, 2009; Sturm et al., 2005) and increased destabilization of permafrost soils (Bonfils et al., 2012; Lawrence & Swenson, 2011). Deciduous shrubs alter soil properties by depositing leaf litter and trapping snow, both of which can buffer ground temperatures, resulting in a deeper active layer and greater nutrient release over winter (Buckeridge & Grogan, 2010; DeMarco, Mack, & Bret‐Harte, 2011; Schimel, Bilbrough, & Welker, 2004). Alternatively, predicted positive feedback effects of shrubs could be offset in summer through increased shading and litter deposition, resulting in shallower active layer depths during the growing season (Blok et al., 2010). Shrubs can also promote herbivore activity, leading to increased shrub sprouting (Tape, Lord, Marshall, & Ruess, 2010) and to changes in hydrology and permafrost thaw associated with predicted beaver encroachment into willow habitat on Alaska's North Slope (Tape, Jones, Arp, Nitze, & Grosse, 2018).

Revegetation following thermal erosion in Arctic and subarctic tundra has resulted in thickets of tall willow, dwarf birch, and alder that persist for decades (Lantz, Kokelj, Gergel, & Henry, 2009; Pizano, Barón, Schuur, Crummer, & Mack, 2014; Schuur, Crummer, Vogel, & Mack, 2007), though in some cases more heterogeneous plant communities develop (Becker, Davies, & Pollard, 2016). The contribution of seedlings versus clonal expansion in forming shrub thickets is not well studied, but thermal erosion appears to provide conditions for increased seed production and suitable microsites for the formation of seedbeds and thickets in some sites (Figure 1c,d; Frost, Epstein, Walker, Matyshak, & Ermokhina, 2013; Lantz et al., 2009). In permafrost regions, deeply thawed mineral soils exposed by RTS formation could allow establishment of deep taproots to anchor seedlings against winter freeze–thaw lifting (Billings & Mooney, 1968), potentially restoring soil stability as plants mature.

Soil seedbanks may be the important sources of colonization where there are sufficient viable seeds and suitable microsites for germination and establishment (Eriksson & Fröborg, 1996). In general, seedbanks are expected to vary in the number and viability of seeds due to differences in seed longevity, standing vegetation, seed production, topography, disturbance frequency, predation, and local climate (Chambers, 1995; Murdoch & Ellis, 1992). In tundra, seedbanks may form under mature vegetation (Fox, 1983) and in depressions and sheltered sites that trap seeds dispersed by wind and water (Alsos, Spjelkavik, & Engelskjøn, 2003; Chambers, 1995). In lower latitudes, high disturbance frequency is predicted to select for seeds that persist in the soil as pioneer species to recolonize uncovered ground (Thompson, 1978). In the Arctic, where disturbance is less frequent and seeds may be more resistant to desiccation (Wyse & Dickie, 2017), seed persistence may be enhanced by burial in the cold, dry conditions of permafrost soils (McGraw, Vavrek & Bennington, 1991). Seedling recruitment in disturbed Arctic soils has been observed from long‐lived buried seed, primarily sedges in the genera *Carex *and *Eriophorum* (Ebersole, 1989; Gartner, Chapin, & Shaver, 1983), and from short‐lived seeds of species not present in the seedbank, including *Betula nana* and *Salix* spp. (Alsos et al., 2003; Cooper et al., 2004; Ebersole, 1989). Species requirements for successful germination and growth may be important in determining whether a site is revegetated by uncovered buried seed or recently deposited seed. The dominance of deciduous shrubs in mature RTS in this area suggests that the source of recruitment is recently deposited seed of species with short‐lived seeds rather than older buried seed. If so, we may expect a random sample of recent postdisturbance seedbanks to contain fewer, mostly viable seeds than older sites.

The goal of this study was to assess (a) whether conditions in RTS are more favorable for seedling recruitment than in tundra undisturbed by RTS and (b) how RTS affect seedbank size and viability. We hypothesized first that, all other things being equal, if recruitment depends upon suitable site conditions, recruitment potential will be higher in RTS than in undisturbed tundra, because disturbed ground is free of competing vegetation and may have more space, light, and available nutrients. Second, we hypothesized that if Arctic seedbanks are more dependent upon nearby mature vegetation than upon long‐distance dispersal and entrapment of seed in depressions, there should be a quantity–quality trade‐off in postdisturbance seedbanks over time, because as seeds accumulate under maturing canopies, the proportion of older seed should increase relative to recent seed input, lowering overall seedbank quality. In contrast, immediately following disturbance, the seedbank may be small in quantity, but high in quality, because it would be composed of mostly recent seed rain. Alternatively, if quantity and quality of Arctic seedbanks are independently influenced by factors such as dispersal, entrapment, seed rain, predation, germination, weathering, disease, and burial, this could result in seedbanks of roughly equal size (i.e., seeds per given area) with different levels of viability or in seedbanks of roughly equivalent viability regardless of size.

We compared environmental conditions across two RTS chronosequences on Alaska's North Slope. We compared in situ seedling counts, seedbank size (seeds m^−2^), and seedbank viability tested in greenhouse germination trials. We predicted that young RTS would have higher in situ seedling counts and smaller but more viable seedbanks. We predicted that due to rebuilding of vegetation canopies and organic layers over time, older RTS would be more similar to undisturbed tundra and show a reverse trend: lower in situ seedling counts and larger seedbanks of lower viability. Finally, we assessed relationships between seedbanks and their environmental conditions to understand whether composition and performance of seedbanks are best explained by RTS age, microsite conditions, location, or some combination of these factors.

## 2 MATERIALS AND METHODS

### 2.1 Study site

**Figure 2:**
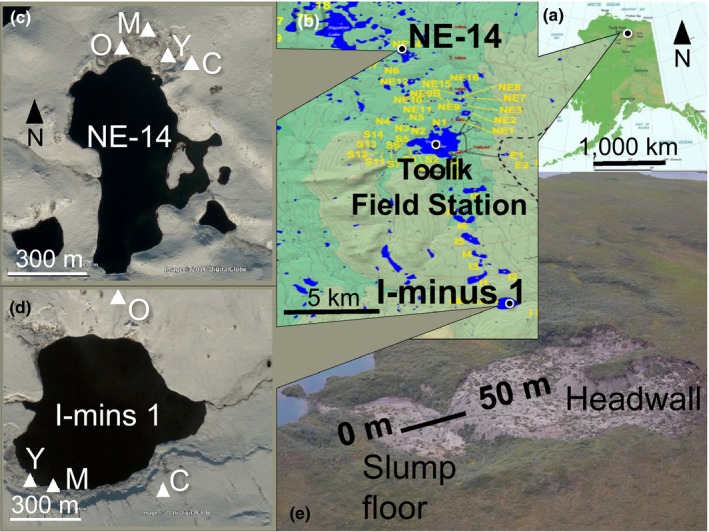
Study sites (a and b) in the Toolik Field Station (TFS) watershed, North Slope Alaska (Maps: Toolik Field Station GIS & Remote Sensing). (c) Sample locations at lake NE‐14 and (d) at lake I‐minus‐1 (Images: Google Earth). Sample locations indicate age of the retrogressive thaw slump (RTS) chronosequences at each lake, abbreviated as Y = young (1–10 years old), M = middle‐aged (11–29 years old), O = old (≥30 years old) and C = undisturbed control outside of the RTS. (e) An example of a 50 m sampling transect used at all sample locations. (Photo credit for (e): Arctic System Science Thermokarst Project)

This study was performed near Toolik Field Station (68°37′39″N, 149°35′51″W), in the northern foothills of the Brooks Range (Figure 2a). The climate is characterized by cold temperatures (−10°C mean annual temperature; 12°C mean July–August temperature), and low precipitation (200–400 mm), nearly half of which falls as snow.

The study area includes mainly moist acidic tussock tundra (MAT), with some areas of heath tundra and shrub tundra. MAT is the most widespread tundra type in the foothills of the Brooks Range, consisting of *Eriophorum vaginatum* and *Carex bigelowii* sedges intermixed with dwarf deciduous shrubs (predominantly *Betula nana* and *Salix pulchra*) and evergreen shrubs (predominantly *Rhododendron tomentosum* Harmaja (1991) and *Vaccinium vitis‐idaea*), herbaceous forbs, mosses (mainly in the genera *Sphagnum, Hylocomium*, and *Aulocomnium*), and lichens (Bliss & Matveyeva, 1992). Heath tundra is found on dry, rocky uplands, and consists of dwarf deciduous (mainly *B. nana*, *Vaccinium uliginosum*, and *Arctostaphylos alpina*) and evergreen shrubs (including *Dryas integrifolia *and/or *D. octopetala,* *V. vitis‐idaea, R. tomentosum*, and *Empetrum nigrum*), with lichens and some mosses. Shrub tundra consists of tall (>0.5 m) thickets of deciduous woody species, including dwarf birch (*B. nana *and/or *B. glandulosa*), shrub willows (*Salix *spp.), some herbaceous forbs and mosses, some pteridophytes (the only pteridophytes we found were horsetails, mainly *Equisetum arvense*) and usually no lichens, normally occurring along water tracks, river gravel bars, and in thermokarst gullies and slumps. All are underlain by continuous permafrost (200 m average depth), with a shallow unfrozen layer that develops in summer (approximately 20–40 cm).

We compared plant recruitment dynamics in undisturbed MAT to that in RTS of different ages around two small lakes (<1 km across), designated as NE‐14 and I‐minus 1 (Figure 2b–d; Pizano et al., 2014). The lakes are approximately 16 km apart on similar slope, aspect, elevation, and parent material. Soils surrounding the lakes are composed of thin peat layers over Itkillik phase II glacial till deposits (approximately 11.5 ka BP; Hamilton, 2003). We chose three RTS age categories at each site: Y = young (1–10 years old); M = middle‐aged (11–29 years old); and O = old (≥30 years old). These were compared with nearby undisturbed MAT at each site (coded as “C” for the undisturbed control condition outside the RTS) for a total of eight study areas, four per site. Age of control areas was undetermined, but they were likely not disturbed by thaw slump thermal erosion for more than 300 years (Pizano et al., 2014). Approximate RTS ages were determined by a previous study through woody shrub growth ring counts and/or radiocarbon dating of moss macrofossils at the organic–mineral soil interface (Pizano et al., 2014). Where feasible, we made shrub ring counts from the same areas and found them to be similar (Table 1).

**Table 1:** Retrogressive thaw slump (RTS) chronosequence sample areas at two sites near Toolik Lake, Alaska. Mean RTS age (±Standard Error) estimated from annual growth ring counts of shrub willow (*Salix* spp.) or dwarf birch (*Betula nana*; *n* shrubs), or estimated from radiocarbon dating of moss macrofossils at the base of the organic soil layer (Pizano et al., 2014)

Site	Transect	Tundra type (dominant plant species)	Mean (±*SE*) RTS age (years)	RTS category	Aging method	*n*
NE‐14	1	Tall shrub (*Salix* spp.)	4.5 (1.1)	Young	Shrub ring counts	16
NE‐14	2	Tall shrub (*Salix* spp.)	25.2 (1.3)^a^	Mid	Shrub ring counts	29^a^
NE‐14	3	Tall shrub (*Salix* spp., *Betula nana*)	30 (1.7)	Old	Shrub ring counts	33
NE‐14	4	MAT (*Eriophorum vaginatum*)	N/A	Control	N/A	N/A
I‐minus 1	1	Sedge‐forb (*Carex* spp., *Epilobium* spp.)	4.3 (0.6)^a^	Young	Shrub ring counts	10^a^
I‐minus 1	2	Tall shrub (*Salix* spp., *Betula nana*)	22.2 (1.3)	Mid	Shrub ring counts	27
I‐minus 1	3	Shrub‐sedge (*Salix glauca, Carex spp.*)	380 (67.2)^a^	Old	Δ^14^C ‰^a^	2^a^
I‐minus 1	4	MAT (*Eriophorum vaginatum*)	N/A	Control	N/A	N/A

Note

MAT: moist acidic tussock tundra. Ages of sample areas in undisturbed controls were not determined (N/A).

a data from Pizano et al. (2014).

The two RTS chronosequences used in this study were analogous in age and type, in close proximity to one another, and were accessible without helicopter support, which we did not have. This low replication reduces our ability to extrapolate to the entire Arctic, but we believe that this study reveals useful information about plant succession following thermal erosion.

### 2.2 Site characterization and observational design

In July 2012 and 2013, we quantified abiotic and biotic characteristics at each site. We chose the month of July in order to quantify site conditions at midsummer, when most seedlings have germinated, plant canopies are fully expanded, and ground is snow‐free. Abiotic characteristics included elevation and soil variables; biotic characteristics included vegetative cover and seedbank variables. Because we expected the greatest seedling recruitment to occur in the lower portion of the RTS where soil had stabilized, in each RTS age category we ran a single 50 m transect on the lower half of the RTS running upslope, with the transect origin (0–1 m) at the downslope end (Figure 2e). Transects in undisturbed controls were located within 500 m of the nearest RTS transect. At each transect, we calculated mean elevation (m) and relative change in elevation (highest point ‐ lowest point) from GPS points to account for RTS depressions versus the flatter ground in undisturbed control locations.

Measurements were made inside 1 × 1 m plots along each transect. We measured soil temperature, soil moisture, and active layer depth at 2.5 m intervals (20 plots per transect), plant cover and seedling counts at 5 m intervals (10 plots per transect), soil nutrients from resin bags (five bags per transect), seed rain traps (five traps per transect), and soil seedbanks (3–4 plots per transect). The locations of resin bag, seed rain, and seedbank plots from transect origin were chosen using a random number generator. Seedbank plots were in the same plots or ≤4 m from abiotic and cover measurements described above and thus represent a subsample of plant cover and in situ seedling plots. Active layer depth, soil temperatures, and soil moisture levels were averaged from three sample points measured inside each plot. Active layer depth was measured using a 1.5 m steel thaw probe driven into the ground until rock or ice was struck. To estimate midsummer soil conditions experienced by seedlings, soil temperature (°C) and percent soil moisture at 5 cm depth were measured using handheld probes. We made visual estimates of aerial percent cover of vascular plants, mosses, and lichens identified to genus or species in each sample plot by counting the number of squares filled by each species using a 1 × 1 m grid divided into 10 × 10 cm squares (optical cramming). In each cover plot, we counted the number of live seedlings (assigning a maximum of 100 seedlings to plots with ≥100 seedlings m^−2^). We recorded the height and width to the nearest 0.1 cm of the tallest shrub and identified shrubs to species or plant functional type. Nomenclature follows Hultén (1968), except where noted. We quantified seed rain by species or functional type by counting the average number of seeds caught over the month of July 2013 in 20 × 20 cm vinyl turf seed traps and dividing by seed trap area (m^2^).

An index of plant‐available soil nutrient levels (NH_4_^+^ and NO_3_^−^) was measured using mixed‐bed ion‐exchange resins (IONAC^®^ nm‐60 H+/OH− form, type I beads 16–50 mesh; J.T. Baker, Phillipsburg, New Jersey, USA). Each resin bag was made of nylon mesh acid‐washed in 10% HCl and rinsed with DI water prior to filling with 9 g fresh weight (fw) of resin, then preloaded with 2 M KCl overnight before being placed into the field. One resin bag per plot was placed in the soil at approximately 5 cm depth, in five randomly selected plots per transect, and left in place from July 6 to 31, 2013. Collected resin bags were transported to the laboratory on ice, washed free of soil using millipore‐filtered water, and stored at −20°C until extraction. Each resin bag was extracted in 100 ml of 2 M KCl agitated overnight on a shaker. Extracts were individually filtered using Whatman grade 1 filter paper, stored at −20°C, and then thawed at 4°C prior to analysis. NH_4_^+^ and NO_3_^−^ concentrations (in μg g^−1^ dw resin) were determined colorimetrically on a Technicon autoanalyzer (Tarrytown, New York, USA) using methods from Whitledge, Malloy, Patton, and Wirick (1981).

### 2.3 Germination experiment

Soil seedbanks (approximately 1 L volume per plot) were taken with a 5 cm diameter × 3 cm depth steel coring tool, 16 cores per plot. Live seedlings found growing in soil cores were added to in situ seedling counts, transplanted to 500 ml pots containing equal parts vermiculite and Promix (Premier Tech, Québec, Canada), and grown for identification to species or functional type in the University of Alaska Research Greenhouse (Fairbanks, Alaska). Soil cores were homogenized for each plot and divided into half. Half of each bulk sample was processed immediately for germination; the other half was frozen at −20°C for 16 weeks before germination (vernalization treatment) in order to induce broad‐spectrum germination of species with different requirements for breaking dormancy (Baskin, Thompson, & Baskin, 2006). In both treatments, we concentrated seedbanks by washing them through 4–0.5 mm mesh soil sieves stacked coarse to fine. This method was developed to optimize germination in native soils by eliminating large particles and fine clays that can create uneven light exposure and moisture conditions (Ter Heerdt, Verweij, Bekker, & Bakker, 1996).

In order to assess germination, a 0.5 cm layer of concentrated seedbank was poured into 16 oz. plastic cups over moistened Whatman #2 filter paper. Cups were incubated in 20 hr daylight/4 hr night at 23.2°C day/21.4°C night to simulate Arctic summer photoperiod. Cups were randomized on benches weekly and inspected weekly for 12 weeks. Germinants were counted and grown until they were large enough to be identified. Ungerminated seeds were air‐dried, counted, and identified to species or functional type in the laboratory under a dissecting microscope. Counts of seeds or germinants m^−2^ were calculated as the mean number of seeds or germinants/core divided by core area (m^2^).

We found willow seeds only in capsules and inferred that single, ungerminated willow seed decomposes rapidly in contact with the soil. We confirmed this in a seed decay experiment by incubating dwarf birch and tall shrub willow seeds collected from branches of live shrubs in a black spruce bog near Fairbanks, Alaska in October 2013. For each species, 100 seeds were placed onto moistened Whatman #2 filter paper in 8 Petri dishes (25 seeds per dish). Petri dishes were covered and incubated in the University of Alaska Research Greenhouse under the above conditions. No additional moisture was added. Initial germination for both species was not different, but after 7 weeks 58% of the willow seed, both germinated and ungerminated, was decayed by mold, versus 3% of the birch seed. We also found wide variation in willow seed counts in our native soil seedbanks, so we pooled birch and willow data for statistical analysis (“birch + willow”). We considered this method appropriate for comparison with other plant functional types because dwarf birch and willow species represented the majority of the deciduous shrub functional type at our sites.

### 2.4 Statistical analysis

To test our hypothesis of the effects of RTS age on each of our dependent variables (in situ seedling counts, seedbank percent germination, and seeds m^−2^), we performed two‐way ANOVA using RTS age categories (four levels): young (Y), middle‐aged (M), old (O), and control (C); site (two levels): NE‐14 and I‐minus 1; and an RTS × site interaction. When the RTS by site interaction was significant, we used one‐way ANOVA to test RTS age effects for each site separately. We performed separate analyses on seedbanks for all species, and for birch + willow. Tukey's Honest Significant Difference test was performed posthoc where effects were significant in ANOVA (*p* < 0.05).

Importance of environmental variables on in situ seedling counts, seedbank percent germination, and seeds m^−2^ was calculated using Akaike's information criterion (AIC) relative importance values for covariates in multiple linear regression models (Akaike, 1992). Because we had many explanatory variables, we considered this a more appropriate method than hierarchical models, because the contribution of any given variable is expressed as a cumulative value across all possible models. For percent germination and seeds m^−2^, the number of observations was too small to evaluate all explanatory variables, and models were ranked using AIC adjusted for the small number of seedbank sample plots (AICc). Importance values were calculated as the cumulative AIC or AICc weight (0 ≤ ∑*ω_i_* ≤ 1; Burnham and Anderson, 2002) using a threshold of ≥0.55 for well‐supported variables (Spellman, Schneller, Mulder, & Carlson, 2015). Environmental variables highly correlated with explanatory variables of interest (Pearson correlation coefficient *r* ≥ 0.60) were omitted from relative importance models and investigated separately in linear regression. Covariates consisted of eleven continuous variables: percent cover of shrubs, forbs, graminoids, nonvascular plants, pteridophytes, litter, and bare soil; percent soil moisture, available NH_4_^+^ and NO_3_^−^; and seed rain m^−2^. Count data were square root transformed using the Box–Cox power transformation (Box & Cox, 1964) to meet assumptions of normal distribution. Distance (m) of each seedling/seedbank plot from its transect origin was tested as a random variable, but was insignificant (*p* > 0.1) and omitted from analysis. Individual data points falling beyond upper and lower quartiles that influenced model coefficients were omitted as outliers (Quinn & Keough, 2002). Due to site interactions on environmental variables, we ran separate relative importance analyses for each chronosequence site (I‐minus 1, NE‐14).

We used canonical correspondence analysis (CCA) to visualize niche separation between species or plant functional types comprising in situ seedlings or soil seedbanks and their respective environmental gradients (Ter Braak, 1986). Count data were standardized to proportions per species or functional type by dividing each sample by its species total, to equalize the contributions of abundant and rare species (Noy‐Meir, Walker, & Williams, 1975). For this analysis, birch and willow seedbanks were analyzed separately. Because we were interested in disturbance as well as environmental effects, sample plots were coded by RTS age class. Evergreen shrub cover was highly correlated with lichen cover (*r* = 0.57) and combined with lichen as heath tundra (“Heath”) for this analysis. Wilk's Lambda (*λ*) was used to test model significance at *p* < 0.05 after 1,000 permutations, and because this statistic is used to report variance not explained by the model, we reported the model effect size as 1 − *λ*, and we reported the variation explained by constrained axes. Where canonical correspondence models were significant, we used ANOVA to determine significance between individual species and environmental variables, and the proportional variance each relationship contributed to the full model. All statistical tests were performed using R 3.4.3 (R Core Team, 2017) with the following packages: agricolae (posthoc tests), MASS (ANOVA), MuMIn (variable importance in multiple regression models), and vegan (canonical correspondence analysis).

## 3 RESULTS

### 3.1 RTS age effects on seedlings and seedbanks

**Figure 3:**
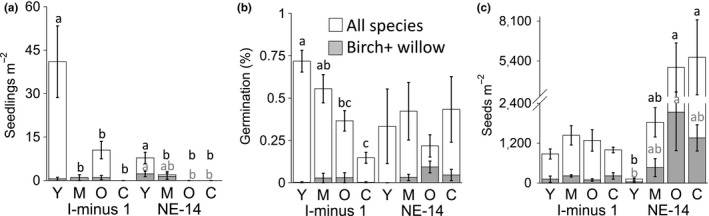
RTS age effects on (a) mean in situ seedling counts m^−2^, (b) seedbank germination in greenhouse trials, and (c) seedbank size (seeds m^−2^) in RTS grouped by site (sites: I‐minus 1 and NE‐14). RTS age abbreviations as in legend to Figure 2. White bars = all species, shaded bars = dwarf birch + shrub willow. Lowercase letters show significant differences between groups in posthoc tests (black letters = all species, gray letters = birch + willow). Error bars show standard error of the mean

There was a significant interaction between RTS age and site for in situ seedlings of all species (Table 2). Although young RTS had the most seedlings at both sites, the young RTS at I‐minus 1 had approximately 5 times as many seedlings as NE‐14 (Figure 3a). In one‐way ANOVA for each site, young RTS had significantly more seedlings than older RTS or undisturbed controls (Table 3). No seedlings were found in undisturbed controls at either site.

**Table 2:** Results of two‐way analysis of variance of in situ seedlings m^−2^, percent germination of soil seedbanks, and seedbank size (seeds m^−2^) by RTS age category (factor levels: Y = Young, M = Middle‐aged, O = Old, C = undisturbed control), site (factor levels: I‐minus 1, NE‐14), and RTS × site interaction

Variables	RTS age					Site					RTS age × site			
	Ndf	Ddf	*F*	*p*	Posthoc	Ndf	Ddf	*F*	*p*	Posthoc	Ndf	Ddf	*F*	*p*
Seedlings m^−2^ a														
All species	3	72	21.742	**<0.001**	Y > O M C	1	72	11.755	**<0.01**	I‐minus 1 > NE‐14	3	72	5.174	**<0.01**
Birch + willow	3	72	3.062	**0.034**	Y > C	1	72	0.687	0.410	NS	3	72	2.135	0.103
Germination (%)														
All species	3	22	2.892	0.057	NS	1	22	1.412	0.247	NS	3	22	2.690	0.071
Birch + willow	3	22	1.792	0.178	NS	1	22	0.732	0.401	NS	3	22	1.465	0.251
Seeds m^−2^ a														
All species	3	22	3.508	**0.032**	C, O > Y	1	22	3.145	0.090	NS	3	22	4.789	**0.010**
Birch + willow^b^	3	22	3.210	**0.043**	C, O > Y	1	22	2.412	0.135	NS	3	22	0.697	0.564

Note

Ddf: denominator degrees of freedom; Ndf: numerator degrees of freedom. Values in bold indicate effects were significant (*p* < 0.05). Posthoc tests (Tukey's HSD) performed where effects were significant at *p* < 0.05. NS is nonsignificant (*p* > 0.1).

a Data were square root transformed to achieve homogeneity of variance.

b Outliers were removed to achieve homogeneity of variance.

**Table 3:** Results of one‐way analysis of variance of in situ seedlings m^−2^, percent germination of soil seedbanks, and seedbank size (seeds m^−2^) by RTS age category (factor levels: Y = Young, M = Mid, O = Old, C = undisturbed control) for each site (sites: I‐minus 1, NE‐14)

Variables	RTS age				
	Ndf	Ddf	*F*	*p*	Posthoc
Site: I‐minus 1					
Seedlings m^−2^ a					
All species	3	36	12.849	**<0.001**	Y > M O C
Birch + willow	3	36	0.501	0.684	NS
Germination (%)					
All species	3	12	15.176	**<0.001**	Y > O C; M > C
Birch + willow	3	12	0.268	0.847	NS
Seeds m^−2^ a					
All species^b^	3	12	1.208	0.349	NS
Birch + willow^b^	3	12	1.017	0.419	NS
Site: NE‐14					
Seedlings m^−2^ a					
All species	3	36	17.059	**<0.001**	Y > M O C
Birch + willow^b^	3	36	4.576	**<0.01**	Y > O C
Germination (%)					
All species	3	10	0.410	0.750	NS
Birch + willow^b^	3	10	2.147	0.158	NS
Seeds m^−2^ a					
All species	3	10	6.170	**0.012**	C, O > Y
Birch + willow^b^	3	10	5.699	**0.015**	C, O > Y

Note

Ddf: denominator degrees of freedom; Ndf: numerator degrees of freedom. Values in bold indicate effects were significant (*p* < 0.05). Posthoc tests (Tukey's HSD) performed where effects were significant at *p* < 0.05. NS is nonsignificant (*p* > 0.1).

a Data were square root transformed to achieve homogeneity of variance.

b Outliers were removed to achieve homogeneity of variance.

Retrogressive thaw slumps age effects were significant for birch + willow seedlings in two‐way ANOVA (Table 2). This is likely because at NE‐14 there were more birch + willow seedlings in the young and middle‐aged RTS but no seedlings in its old RTS or undisturbed control (Table 3); I‐minus 1 birch + willow seedling counts were not different (Figure 3a). Although birch + willow counts were low, they comprised the majority of in situ seedlings in middle‐aged RTS at both sites (Figure 3a).

Retrogressive thaw slumps age affected germination and size of seedbanks independently at the different sites. There was a marginally significant interaction between RTS age × site for percent germination (Table 2), because the young I‐minus 1 seedbank showed 2–5 times greater percent germination than its old and undisturbed control seedbanks (Figure 3b); percent germination of NE‐14 seedbanks was not different (Table 3; Figure 3b). For both sites, birch and willow germination was low, between 1% and 6% (Figure 3b), and not different (Table 3). RTS age × site interaction was significant for seedbank size (Table 2), because NE‐14 seedbanks ranged from 122 ± 34 seeds m^−2^ (young) to 5,651 ± 2,538 seeds m^−2^ (undisturbed control); I‐minus 1 seedbanks were not different (Table 3; Figure 3c). There was no RTS age × site interaction for birch + willow seedbank size (Table 2), but RTS age was significant for birch + willow seedbanks in two‐way ANOVA (Table 2), likely driven by the larger seedbanks at NE‐14 (Figure 3c). Birch + willow comprised 25%–50% of NE‐14 seedbanks, ranging from 27 to over 4,300 seeds m^−2^ across the chronosequence, versus 30–400 seeds m^−2^ at I‐minus 1 (Figure 3c).

### 3.2 Environmental conditions and site characterization

**Figure 4:**
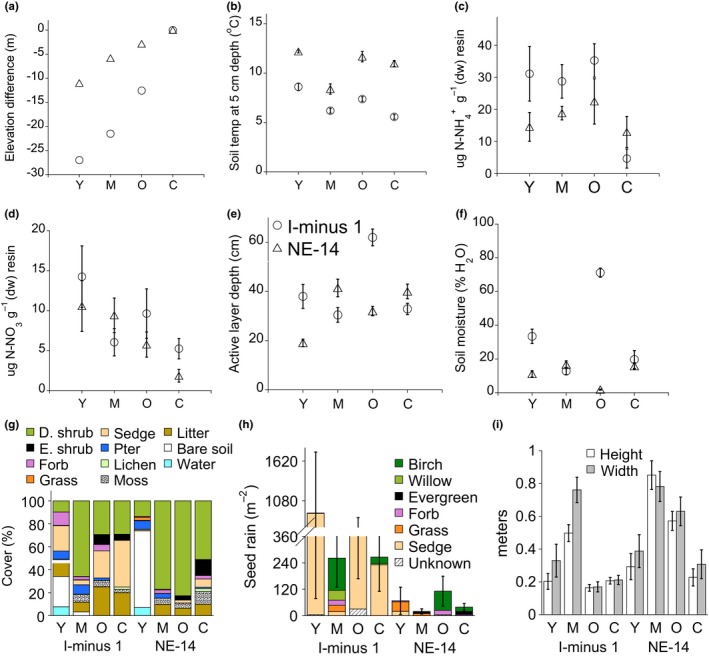
Environmental conditions at two retrogressive thaw slump (RTS) chronosequence sites (sites: I‐minus 1, NE‐14): (a) Elevation difference between the highest and lowest elevation at each site, (b) soil temperature at 5 cm depth, (c) available soil ammonium, (d) available soil nitrate (c and d assayed with resin bags), (e) active layer depth, (f) percent soil moisture at 5 cm depth, (g) percent cover of vegetation, (g) seed rain counts, and (i) mean height and width of tallest shrub. Error bars show standard error of the mean. RTS age abbreviations as in legend to Figure 2. Circles = sample locations at lake I‐minus 1, triangles = sample locations at lake NE‐14. Percent cover: Birch = *Betula* *nana*, D. shrub = deciduous shrubs, E. shrub = evergreen shrubs, Forb = herbaceous forbs, Grass = *Arctagrostis*, *Calamagrostis*, and *Poa *spp*.*, Pter = pteridophytes (*Equisetum* spp.), Lichen = live lichens, Moss = live mosses, Litter = litterfall, Sedge = *Carex* and *Eriophorum* spp. Willow = *Salix* spp., Unknown = not identified to species or functional type, Soil = bare mineral soil, Water = standing water

Environmental conditions were different in RTS than in undisturbed tundra. RTS sampled on the same hillslopes were more collapsed and lower in elevation than undisturbed controls (Figure 4a). Midsummer soil temperatures at I‐minus 1 were 2°C warmer in its young RTS than in other age categories; at both sites, the middle‐aged RTS were among the coolest (Figure 4b). Soil available NH_4_^+^ at I‐minus 1 was six times higher in RTS than undisturbed (Figure 4c), and available NO_3_^−^ was three to five times higher in young versus undisturbed at both sites (Figure 4d). Similar to other studies (Bonfils et al., 2012; Lantz et al., 2009), active layer depths were deeper in older RTS than in undisturbed (I‐minus 1, Figure 4e), but we excluded active layer depth from our analyses due to surface rubble in young RTS impeding probes from reaching the frozen layer. Percent soil moisture varied from 1.9% ± 0.3% at NE‐14 to 71% ± 3% at I‐minus 1 and was not associated with RTS age (Figure 4f).

Percent cover showed RTS age effects common to both sites. Young RTS had more bare soil, middle‐aged RTS were dominated by tall deciduous shrubs, and older and control plots featured more evergreen shrubs and lichens (Figure 4g). Sites showed local differences in cover and seed rain composition: NE‐14 had more evergreens, and I‐minus 1 had more sedges and forbs (Figure 4g,h). Total seed rain was not different between sites, with dwarf birch and willow seed comprising about 10% (Figure 4h). The largest shrubs, mainly willows and dwarf birch, were found in middle‐aged RTS at both sites and were on average nearly 0.5 m taller and wider than shrubs in other RTS age groups (Figure 4i).

### 3.3 Relative importance of environmental characteristics

**Figure 5:**
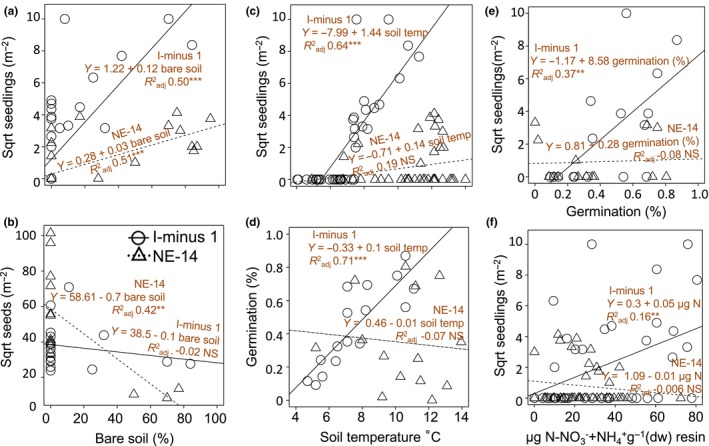
Regression relationships between seedling or seedbank variables and explanatory variables (a) in situ seedling counts as a function of percent bare soil, (b) seedbank density as a function of percent bare soil, (c) in situ seedling counts as a function of soil temperature at 5 cm depth, (d) percent germination of seedbanks as a function of in situ soil temperature, (e) in situ seedling density as a function of greenhouse (percent) germination of soil seedbanks, and (f) in situ seedling density as a function of available nitrogen (NH_4_^+^ and NO_3_^−^ pooled for analysis). Circles with unbroken trend line: sample locations at lake I‐minus 1; triangles with dotted trend line: sample locations at NE‐14. Insets show model parameters. Count data were square root transformed to meet assumptions of normal distribution

Bare soil had high relative importance at both sites and occurred in over 93%–99% of all possible regression models using the parameters in Tables 4 and 5 to explain the variation in in situ seedlings at both sites (Tables 4 and 5). In situ seedling counts increased by 0.06 and 0.03 seedlings m^−2^ for every unit increase in bare soil at I‐minus 1 and NE‐14, respectively (Tables 4 and 5). By itself, bare soil explained over 50% of variance in in situ seedling counts at each site in linear regression (*F*_1,38_ = 40.72, *p < *0.0001 at I‐minus 1 and *F*_1,38_ = 41.46, *p < *0.0001 at NE‐14; Figure 5a). Bare soil was important in explaining higher birch + willow seedling counts and smaller seedbanks at NE‐14 (Table 5). By itself, bare soil explained 42% of the variation in seedbank size at NE‐14 in linear regression (*F*_1,12_ = 10.39, *p < *0.001; Figure 5b).

**Table 4:** Modeled Akaike's information criterion (AIC) average parameter estimates (*b*) and relative variable importance expressed as cumulative parameter weights (0 ≤ ∑*ω_i_* ≤ 1) for variables explaining differences in in situ seedling counts m^−2^, seedbank % germination, and seedbank size (seeds m^−2^) at I‐minus 1

Site: 1‐minus 1							
Species	Explanatory variables	Response variables					
		In situ seedlings m^−2^		Germination (%)		Seeds m^−2^	
		$\overset{¯}{b}$	∑*ω_i_*	$\overset{¯}{b}$	∑*ω_i_*	$\overset{¯}{b}$	∑*ω_i_*
All species	Shrub cover	−0.06	0.93	−0.01	0.89	–	0.39
	Bare soil	0.06	0.92	–	0.16	–	0.13
	Graminoid cover	−0.07	0.86	−0.01	0.92	–	0.12
	Soil moisture	0.01	0.57	–	0.07	–	0.11
	NH_4_^+^	–	0.51	–	–	–	–
	Nonvascular plant cover	–	0.43	–	0.09	–	0.18
	Litter	–	0.26	–	0.10	–	0.12
	Pteridophytes	–	0.25	0.02	0.56	–	0.23
	NO_3_^−^	–	0.20	–	0.08	–	0.20
	Seed rain	–	0.18	–	0.07	–	0.43
	Forb cover	–	0.18	–	0.11	–	0.13
Birch + willow	Bare soil	–	0.44	–	0.14	–	0.13
	Nonvascular plant cover	–	0.38	–	0.26	–	0.20
	Pteridophytes	–	0.38	–	0.17	–	0.11
	NH_4_^+^	–	0.34	–	–	–	–
	NO_3_^−^	–	0.33	–	0.16	–	0.16
	Soil moisture	–	0.32	–	0.12	–	0.14
	Litter	–	0.32	–	0.12	–	0.23
	Graminoid cover	–	0.27	–	0.20	–	0.12
	Shrub cover	–	0.23	–	0.20	–	0.16
	Forb cover	–	0.21	–	0.22	–	0.43
	Seed rain	–	0.20	–	0.13	–	0.26

**Table 5:** Modeled Akaike's information criterion (AIC) average parameter estimates (*b*) and relative variable importance expressed as cumulative parameter weights (0 ≤ ∑*ω_i_* ≤ 1) for variables explaining differences in in situ seedling counts m^−2^, seedbank % germination, and seedbank size (seeds m^−2^) at NE‐14

Site: NE‐14	Explanatory variables	Response variables					
Species		In situ seedlings m^−2^		Germination (%)		Seeds m^−2^	
		$\overset{¯}{b}$	∑*ω_i_*	$\overset{¯}{b}$	∑*ω_i_*	$\overset{¯}{b}$	∑*ω_i_*
All species	Bare soil	0.03	0.99	–	0.08	−0.63	0.79
	Soil temperature	–	0.21	–	0.08	–	0.09
	Shrub cover	–	0.21	–	–	–	–
	Graminoid cover	–	0.34	–	0.18	–	0.09
	Soil moisture	–	0.26	–	0.35	–	0.18
	Nonvascular plant cover	–	0.21	–	0.14	–	0.24
	NH_4_^+^	–	0.24	–	0.11	–	0.27
	Litter	–	0.34	–	0.10	–	0.09
	Seed rain	–	0.22	–	0.50	–	0.08
	Pteridophytes	–	0.22	–	0.22	–	0.17
	Forb cover	–	0.20	–	0.09	–	0.11
	NO_3_^−^	–	0.26	–	0.27	–	0.10
Birch + willow	Bare soil	0.01	0.71	–	0.53	−0.42	0.70
	Litter	–	0.37	–	0.21	–	0.07
	Shrub cover	–	0.36	–	–	–	–
	NO_3_^−^	–	0.35	–	0.08	–	0.25
	Soil moisture	–	0.24	−0.004	0.71	−0.98	0.56
	Forb cover	–	0.23	–	0.09	–	0.08
	Nonvascular plant cover	–	0.22	–	0.08	–	0.08
	Pteridophytes	–	0.22	–	0.12	–	0.30
	Seed rain	–	0.22	–	0.23	–	0.08
	Soil temperature	–	0.21	–	0.18	–	0.09
	NH_4_^+^	–	0.20	–	0.07	–	0.08
	Graminoid cover	–	0.20	–	0.13	–	0.10

Shrub and graminoid cover were dominant at I‐minus 1, and of high relative importance at this site. Seedlings and percent germination decreased on average by −0.07 and −0.01, respectively, per unit increase in shrub and graminoid cover (Table 4). Available NH_4_^+^ and NO_3_^−^ had low relative importance in models explaining in situ seedlings at I‐minus 1 (Table 4), but in linear regression they explained 16% of the variance in in situ seedlings (*F*_1,38_ = 8.61*, p* < 0.01; Figure 5f).

### 3.4 Other environmental conditions related to bare soil

We looked at environmental variables that had to be omitted from relative importance analysis due to their high correlation with bare soil. Soil temperature was highly correlated with bare soil at I‐minus 1 (*r* = 0.78); by itself, it explained 64% of the variation in in situ seedling counts in linear regression (*F*_1,38_ = 71.02, *p < *0.0001; Figure 5c). Percent germination of I‐minus 1 seedbanks in the greenhouse increased linearly for samples taken from plots with soil temperatures ranging from 5 to 11°C (Figure 5d), accounting for 71% of model variance in linear regression (*F*_1,14_ = 36.88, *p < *0.0001). In situ seedling abundance was highly correlated with bare soil at I‐minus 1 (*r* = 0.75) and was useful to understand recruitment dynamics at this site. When percent germination in the greenhouse was used to predict in situ seedling counts in linear regression, the model was significant, explaining 37% of the variance in seedling abundance at I‐minus 1 (*F*_1,14_ = 9.83*, p* < 0.01; Figure 5e), demonstrating that seedling recruitment may be occurring from this seedbank.

### 3.5 Niche separation of seedlings versus seedbanks

**Figure 6:**
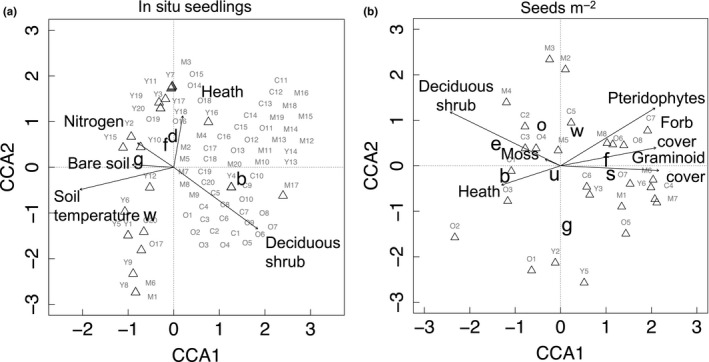
Canonical correspondence ordination plots of retrogressive thaw slump sites show relationships between species or plant functional type (lowercase letters) comprising seedlings or seedbanks and environmental variables including plant functional type cover (arrows labeled with uppercase type) for (a) in situ seedlings m^−2^ and (b) seedbanks (seeds m^−2^). Plot IDs (in gray) are expanded around centroids (triangles) for clarity. Arrow length indicates strength of environmental gradient. Proportional variability in weighted regression analysis explained by first two axes in *n* = 1,000 permutations is significant in (a) (*p* < 0.01) and (b) (*p* < 0.001). Species of seedlings and seeds: b = *Betula nana*, d = unidentified dicots, f = forbs, e = evergreens, g = graminoids, o = other deciduous species, s = sedge, u = unidentified, w = shrub willow. “Heath” is pooled percent cover of lichens and evergreen shrubs, and “Nitrogen” is pooled available NH_4_^+^ and NO_3_^−^. Plot numbers are preceded by RTS age category abbreviated as in Figure 2

Canonical correspondence plots suggest there may be less niche separation of species at the recruitment stage than during formation of seedbanks (Figure 6a,b). In situ seedlings of most species were more abundant with bare soil and available nutrients common to young RTS (Figure 6a). The full model explained half of the variation and was significant in MANOVA (Wilk's Lambda (*λ*): 0.467, *F*_25,__261.54_ = 2.383, *p* < 0.001). The model produced four correlation functions between five species and five environmental gradients, with most correlations explained in the first two axes (constrained eigenvalues: 0.621, 0.391, 0.287, and 0.148 for CCA1 through CCA4, respectively). Linear Combination (LC) scores of CCA 1 described a gradient of undisturbed and older RTS sites: greater shrub cover, less bare soil, lower soil temperatures, and less available nitrogen, where most birch seedlings were found. CCA2 described a gradient of open ground containing forb, graminoid, and dicot seedlings, less shrub cover, warmer soils, and more available nitrogen characteristic of young (“Y”) RTS plots and some heath characteristic of late‐succession and undisturbed control plots (“O” and “C,” respectively; Figure 6a). In posthoc univariate analysis, graminoid and forb seedlings were, respectively, associated with bare soil and available nitrogen, accounting for 21% and 14%, respectively, of model variance in ANOVA (*F*_5,74_ = 5.27, *p* < 0.001 and *F*_5,74_ = 3.56, *p* < 0.01, respectively; Figure 6a). Willow seedling counts were significantly higher in bare soil plots in linear regression (*F*_1,78_ *= *5.40, *p* = 0.022), but canonical plots show a trend of willow seedling abundance with warmer soils and decreasing heath cover, explaining 7% of model variance in ANOVA (*F*_5,74_ = 2.15, *p* = 0.069; Figure 6a). Birch and unidentified dicots showed no significant association to environmental gradients in ANOVA (*F*_5,74_ = 0.471, *p* = 0.797 and *F*_5,74_ = 0.520, *p* = 0.76, respectively), likely due to scattered distribution across gradients.

Seedbank sizes (seeds m^−2^) were larger with mid‐ to late‐succession and undisturbed control plant cover, including cover of the same species or plant functional type (Figure 6b). The full model produced six correlation functions for eight species and five environmental variables (eigenvalues for constrained axes 1–6: 0.279, 0.189, 0.127, 0.064, 0.035, and 0.002, respectively) and was significant in MANOVA (Wilk's *λ*: 0.029, *F*_48,__82.789_ *= *1.816, *p* < 0.01). Variance explained by constrained axes was 44%, about half of the variance expressed as 1 − *λ*. LC scores in CCA1 described a gradient of tundra dominated by graminoids, forbs, and pteridophytes; CCA2 explained a gradient of deciduous shrub tundra with pteridophytes, some forbs and mosses, and heath cover. In univariate analysis, sedge seedbanks were significantly larger with increasing graminoid, forb, and pteridophyte cover (ANOVA: *F*_6,23_ *= *3.94, *p* = 0.008; Figure 6b), explaining 38% of model variance. Forb seedbanks were larger with greater graminoid, moss, and pteridophyte cover, and smaller with increasing heath cover, accounting for 32% of the variance in ANOVA (*F*_6,23_ *= *3.28, *p* = 0.018; Figure 6b). Willow seedbanks showed a trend of larger size with deciduous shrub and forb cover in ANOVA (*F*_6,23_ *= *2.34, *p* = 0.065; Figure 6b), as did evergreen seedbanks (mainly *Vaccinium* spp. and *Empetrum nigrum*) with mosses (*F*_6,23_ *= *2.284, *p* = 0.071; Figure 6b), explaining 22% and 21% of model variance, respectively. Seedbanks of birch and “other deciduous” species (mainly *Arctostaphylos* spp.) were somewhat positively associated with greater deciduous shrub cover although these results were of little significance in ANOVA (*F*_6,23_ *= *2.14, *p* = 0.087 and *F*_6,23_ *= *1.78, *p* = 0.142, respectively). Grass seedbanks had no significant relationship to cover (ANOVA: *F*_6,23_ *= *0.79 *p* = 0.59), likely due to spatially scattered distribution.

## 4 DISCUSSION

### 4.1 RTS and seedbank dynamics

We predicted seedling recruitment was higher in RTS than in surrounding undisturbed tundra and best in young RTS due to a newer seedbank and better conditions for germination and growth. We found in situ seedlings only in RTS; no seedlings were found in undisturbed tundra, lending support for our hypothesis. We found no evidence of a trade‐off between seedbank quantity and quality to support our second hypothesis. Age effects on in situ seedling counts and greenhouse germination trials demonstrate that the source of recruitment in some RTS is likely from the seedbank. Our results suggest that recent RTS can be potential hotspots of seedling recruitment and recruitment rates appear to be comparable to other types of tundra disturbance (Nystuen et al., 2014; Sutton et al., 2018).

### 4.2 Role of environmental characteristics in recruitment

Seedling success in Arctic tundra has been associated with the absence of neighboring plants (Gough, 2006), germination on bare soil (Billings & Mooney, 1968; Bishop & Chapin, 1989; Noble, 1979; Van Splunder, Coops, Voeseneck, & Blom, 1995), warmer soils (Milbau, Graae, Shetsova, & Nijs, 2009), moisture (Bell & Bliss, 1980), nutrients (Gough, Bass, & McLaren, 2015), shelter provided by plants (Billings & Mooney, 1968; Carlsson & Callaghan, 1991; Cooper et al., 2004; Graae et al., 2011), and depressions in the ground (Alsos et al., 2003; Graae et al., 2011). Our in situ seedling counts and seedbank viability were either negatively correlated with plant cover or had no association. We found bare soil to be a variable of high importance, likely because it integrates other variables of biological significance, including space for germination and warmer soils. We did not measure RTS sheltering effects but found warmer soil temperatures in young RTS plots, which tended to be more collapsed than undisturbed ground. Sheltered microsites may reduce exposure of seeds and seedlings to winds in the lee of the RTS headwall or buffer temperature extremes by trapping snow (Sturm et al., 2001), allowing rapid germination following snowmelt. Conversely, winter seedling mortality may be higher in sheltered depressions where seedling emergence is high (Graae et al., 2011; Venn & Morgan, 2009); however, sites of high average recruitment may experience higher seedling turnover (Van Mantgem, Stephenson, & Keeley, 2006). Although seedling niches may vary among species (Eriksson, 2002), our canonical correspondence analysis shows seedling abundance across species and plant functional types was greater in recently disturbed ground where soils are more likely to be bare, warmer, and more nutrient‐rich than late‐succession or undisturbed tundra.

Increased thawing is predicted to change the nutrient balance of permafrost soils, resulting in vegetation shifts as tundra communities respond to increased nutrient availability (Becker et al., 2016; Gooseff et al., 2009; Wang et al., 2017). Fertilization studies in the Toolik Lake area demonstrated that nutrients stimulate deciduous shrub growth (Chapin, 2005), and when nutrient demands are met, shrub productivity becomes more sensitive to other limiting factors such as temperature and light availability (Shaver et al., 2001). In open, warm microsites with adequate nutrient supply, dwarf birch and willow recruits can be expected to overtop other species within a few years. Compared to our undisturbed controls, available NH_4_^+^ did not change in RTS soils of different disturbance age, possibly due to the effects of higher quality litter production in older shrub‐dominated RTS (Buckeridge, Zufelt, Chu, & Grogan, 2009). Available NO_3_^−^ showed a similar pattern to differences in soil temperature at the middle‐aged RTS at I‐minus 1 (Figure 4d,b). A decrease in NO_3_^−^ levels within the first 1–2 decades at the middle‐aged RTS implies plants are taking up the available nutrient supply. The fact that we found cooler soils under the tallest and widest shrubs suggests the combined effects of leaf canopy shading and litterfall. These results agree in part with previous research that shrubs may affect ground temperatures differently in summer than in winter (Blok et al., 2010).

### 4.3 Seedbank characteristics

The lack of a trade‐off between seedbank size and viability over time in our results suggests these properties are independent. Seedbanks in open tundra environments are expected to form through entrapment of wind‐ and water‐dispersed seed (Alsos et al., 2003; Chambers, 1995) and from seed rain (Fox, 1983). Although we found RTS to be more collapsed than adjacent undisturbed areas, we found no evidence that RTS sites trapped more seed than the flatter ground of undisturbed tundra. RTS are destructive events, resulting in soil wasting and mixing of soil layers (Pizano et al., 2014), and may be composed of combinations of new seed rain and uncovered old seed (Ebersole, 1989; Gartner et al., 1983; McGraw et al., 1991). In partial support of Fox's (1983) prediction that seedbank size increases with plant productivity and seed rain, we found most seedbanks associated with plant cover, although seed rain showed no relationship to seedbank size. Our seedbanks ranged from 71 to 10,000 seeds m^−2^ (untransformed count data) and were not different from seedbank sizes reported in studies from temperate and northern latitudes (Alsos et al., 2003; Cooper et al., 2004; Ebersole, 1989; Fox, 1983; Thompson, 1978). The fate of Arctic seeds is therefore likely determined by the same interactive processes that form seedbanks at other latitudes (Chambers, 1995; Thompson, 1978).

In contrast to sedge‐dominated seedbanks found in some Alaskan Arctic soils (Ebersole, 1989), the largest seedbanks we found were from NE‐14’s old RTS and undisturbed tundra and were primarily composed of dwarf birch (*Betula nana*) and evergreen species (mainly *Vaccinium* spp. and *Empetrum nigrum*). This may be in part due to our goal to sample relatively recent seed input at an average depth of 3 cm, compared to Ebersole's (1989) sampling at 10 cm depths, as the latter method likely resulted in greater numbers of buried seed. Dwarf birch seed is common throughout the Arctic, but is considered short‐lived compared to sedge species, many of which are known to survive burial (Ebersole, 1989). Because short‐lived seed may not be adequately represented in studies of soil seedbanks at deeper depths, we felt our sampling method was appropriate in order to understand the relationship of seedbanks to the tall shrub thickets we found in RTS.

Birch and willow seed accounted for over half of some seedbanks, but low percent germination along with higher decay of willow seed in our greenhouse trials suggests recruitment in the field may be lower. Seedbank studies of High Arctic populations (Cooper et al., 2004) suggest greenhouse trials may not reflect in situ recruitment; however, Low Arctic populations may not be as seed‐limited as High Arctic populations due to a longer growing season, likely resulting in greater production of viable seed. Ebersole (1989) found dwarf birch and willow are common colonizers of disturbed tundra in the Alaskan Low Arctic, and given that birch and willow produce large quantities of seed, in situ germination rates of 1%–6% could be sufficient for recruitment in suitable microsites. It has been estimated that as few as 6–38 seedlings, followed by clonal growth, is sufficient to establish existing populations of these species at the northern limits of their ranges (Alsos et al., 2007). Perhaps the best evidence that germination rates may be sufficient for shrub establishment in the Toolik Lake area is that the RTS we observed were filled with shrubs within a few decades.

We did not follow the fate of in situ seedlings over time, nor did we account for resprouting vegetative propagules in this analysis (Alsos et al., 2003). Evidence from temperate and boreal forest fires suggests that seedling counts of woody species are highest within the first 5 years of disturbance and that revegetation occurs within the first decade (Johnstone et al., 2004; Romme, Turner, Tuskan, & Reed, 2005; Rydgren, Økland, & Hestmark, 2004). Similarly, thermokarst revegetation can occur if exposed soils stabilize within a year after disturbance (Gooseff et al., 2009). Our results agree qualitatively with these, because we saw the most seedlings in the sites aged to within 1 decade old.

### 4.4 Tundra succession following RTS

It is unknown whether increasing frequency of thermal erosion will lead to different vegetation communities (Becker et al., 2016; Wang et al., 2017) or if the tall shrub thickets we see in RTS in the Toolik Lake area represent a mid‐successional stage of MAT recovery. Viereck (1966) states that the transition from tall deciduous shrub thickets to dwarf shrubs, and finally to MAT, is more likely to occur once moss layers are developed, as this allows lateral expansion of adventitious roots for species other than willow, and creates suitable moisture regimes for establishment of *E. vaginatum *tussocks. Ages of the two oldest RTS we sampled estimated in a previous study were found to be widely different (30 years and 380 years, respectively; Table 1), but these differences are useful in understanding what might happen decades versus centuries after RTS formation. Average canopy height of the tallest shrubs we measured at the 30‐year‐old RTS was over 0.4 m taller than at the 380‐year‐old RTS (Figure 4i). Interestingly, 3–4 centuries after disturbance, vegetation at the oldest RTS (at I‐minus 1) was more similar in height and composition to the undisturbed MAT control located on the opposite side of the lake at this chronosequence (Figures 2d and 4g,i). This and previous studies of the area suggest that although disturbance type and severity may influence successional outcomes, tundra communities appear to be resilient (Bret‐Harte et al., 2013; Vavrek et al., 1999). Our observations at I‐minus 1 and NE‐14 suggest that tall shrub thickets may not represent a vegetation shift, but rather a mid‐successional phase, and that MAT may require several centuries to recover from RTS disturbance.

### 4.5 Adaptive potential of seedbanks to climate change

Seedbanks represent a naturally occurring genetic time capsule of a past world. The cold dry conditions of permafrost soils may be similar to artificial seedbanks in preserving seed longevity (Yashina et al., 2012); however, it is largely unknown how old viable buried seed may be or to what extent it contributes to Arctic vegetation communities. Historically, the Arctic has transitioned from graminoid tundra to shrub tundra in response to changing climate patterns during the Late Glacial Maximum (Mann, Groves, Kunz, Reanier, & Gaglioti, 2013; Naito & Cairns, 2011), but it is not well understood whether new populations arising from long‐buried seed can adapt to rapid anthropogenic change. Successful germination of plants from putative ancient seedbanks (Yashina et al., 2012) and genetic diversity comparisons of above‐ground populations to their seedbanks (Honnay, Bossuyt, Jacquemyn, Shimono, & Uchiyama, 2008) suggest that species with long‐lived seed may have sufficient genetic resources to adapt to rapid change. Species producing short‐lived seed, on the other hand, may be at greater risk of extinction through genetic drift if external changes lead to habitat loss (Honnay et al., 2008). Shrub expansion in disturbed permafrost soils may be especially critical for species that produce ephemeral seed, as they may rely more heavily on recruitment and establishment to maintain gene flow than species with persistent seed.

Although it is likely that thermokarst failures are occurring with greater frequency now than in the past, lake sediments and charcoal deposits provide evidence of Late Holocene thermokarst activity 3,000–10,000 years ago in the Canadian Arctic (Dallimore, Schröder‐Adams, & Dallimore, 2000) and Siberia (Katamura, Fukuda, Bosikov, & Desyatkin, 2009). It has been proposed that due to low rates of plant turnover in the Arctic, the plants we see today and their seedbanks are not very different genetically from historical populations, and that adaptation to future conditions may be dependent upon recruitment of new populations (McGraw, 1993). Our study found that thermal erosional disturbance may stimulate recruitment in an area of the world where the contribution of seedlings is considered infrequent.

## 5 CONCLUSION

Our work suggests that shrub recruitment from seed in young RTS could be important in the development of the tall shrub communities we observed in older RTS. As with previous studies, our study has uncovered the importance of local variation in environmental characteristics as potential predictors of seedling success in the Arctic. Additional research of recruitment and thermal erosion at additional sites is needed, but our results suggest that due to opportunities for seed germination in nutrient‐rich open ground and to potential sheltering effects, RTS may act as seedling nurseries that could benefit many Arctic species, especially those which do not produce persistent seed.

## CONFLICT OF INTEREST

None declared.

## AUTHOR CONTRIBUTIONS

DCH and MSBH conceived of the ideas and designed the methodology; DCH conducted field sampling and greenhouse experiments. DCH analyzed the data, with assistance from MSBH and others. Both authors contributed critically to the drafts and gave final approval for publication.

## Data Availability

All data will be archived in the Dryad Digital Repository. Provisional DOI: https://doi.org/10.5061/dryad.rh807jp Data files: Seedling and Seedbank data.
